# Supplementary material for: Pilot Study of Anti-Th2 Immunotherapy for the Treatment of Breast Cancer-Related Upper Extremity Lymphedema
Source: Biology (Basel). 2021 Sep 18;10(9):934. doi: 10.3390/biology10090934 (PMC8466465; doi:10.3390/biology10090934)
Supplement: Supplementary file 1 [file biology-10-00934-s001.zip › supplimentary figures.pdf]

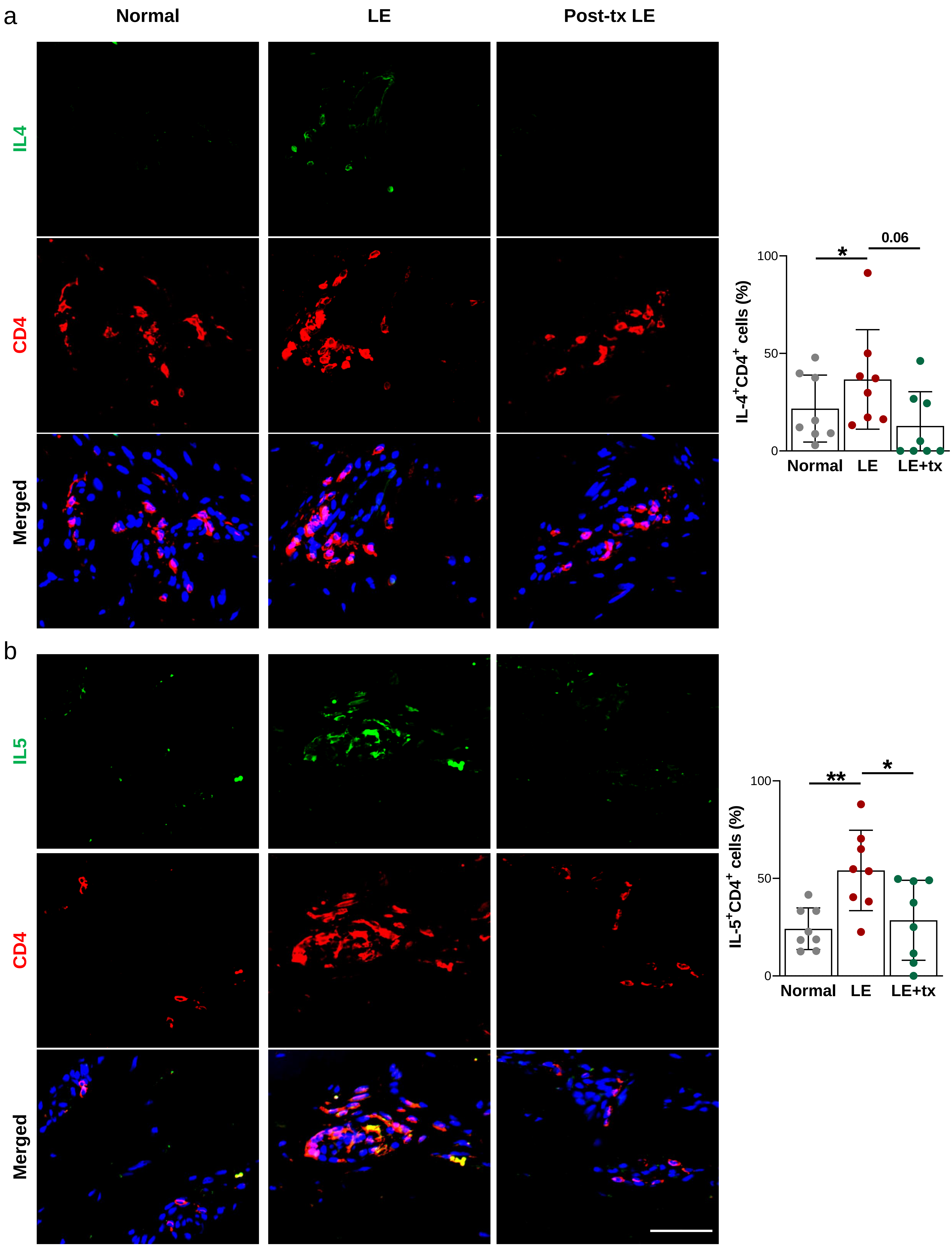
a
Normal
LE
Post-tx LE
IL4
CD4
Merged
IL-4+CD4+ cells (%)
100
50
0
*
0.06
Normal
LE
LE+tx
b
IL5
CD4
Merged
IL-5+CD4+ cells (%)
100
50
0
**
*
Normal
LE
LE+tx

Supplementary Figure 1. Treatment with QBX258 decreases the number of putative Th2 cells in lymphedema biopsy specimens. **Scale bar: 200 μm; P* < 0.05; P** < 0.01.**

a. Representative immunofluorescent staining (left panels) and quantification (right panel) of IL4 (green, top panels), CD4 (red), DAPI (blue) merged images in normal, LE, LE+tx biopsy specimens.

b. Representative immunofluorescent staining (left panels) and quantification (right panel) of IL5 (green, top panels), CD4 (red), DAPI (blue) merged images in normal, LE, LE+tx biopsy specimens.
